# Clozapine Induced Pericarditis: A Systematic Review

**DOI:** 10.1192/bjo.2025.10248

**Published:** 2025-06-20

**Authors:** Aliu Yakubu, Olorungbami Anifalaje, Moses Effiong, Oluwakemi Olalude, Maryam Abubakar

**Affiliations:** 1 University Hospital Wishaw, Wishaw, United Kingdom; 2 NHS Dumfries and Galloway, Dumfries, United Kingdom; 3 Glasgow Caledonian University, Glasgow, United Kingdom; 4 Lagos State University Teaching Hospital, Ikeja, Nigeria

## Abstract

**Aims:** Clozapine is an atypical antipsychotic for treatment-resistant schizophrenia. Despite its efficacy, there are potential life-threatening side effects, including pericarditis, which has limited its usage. Clozapine-induced pericarditis may range from mild symptoms to life-threatening complications. Despite increasing case reports, a comprehensive synthesis is lacking, necessitating a systematic review.

**Methods:** A systematic review was conducted following PRISMA 2020 guidelines and registered in PROSPERO. Eight databases, including PubMed, Embase, and PsycINFO, were searched, identifying case reports published between 1980 and 2024. Inclusion criteria focused on English-language case reports diagnosing clozapine-induced pericarditis. Exclusion criteria included non-clozapine-induced pericarditis and mixed aetiologies without clozapine-specific data. Data extraction included demographics, clinical presentation, diagnostic findings, management, and outcomes.

**Results:** Of the 941 identified articles, 36 met the inclusion criteria. The mean age was 33.56 years (SD: 15.56), with males comprising 63.9%. Chest pain (63.8%), fever (52.8%), breathlessness (50%), and tachycardia (44.4%) were the most common symptoms. Diagnostic tests consistently indicated elevated inflammatory markers, including CRP (mean: 88.13 mg/dL) and ESR (mean: 72.72 mm/hr). Echocardiograms confirmed pericardial effusion in 88.9% of cases. Management strategies included colchicine (16.7%) and analgesics (19.4%), with cardiac recovery achieved in all but one case. Clozapine rechallenge was attempted in 16.7%, with successful outcomes in 83.3% of these cases. Time to recovery averaged 3.73 weeks (SD: 9.8). Psychiatric stability was maintained in most cases following substitution with alternative antipsychotics, primarily olanzapine and risperidone.

**Conclusion:** Clozapine-induced pericarditis is a rare but significant adverse event characterized by elevated inflammatory markers and diagnostic imaging abnormalities. Prompt recognition and tailored management, including anti-inflammatory treatment and careful rechallenge, can lead to favourable cardiac and psychiatric outcomes. This review underscores the need for heightened clinician awareness and standardized protocols to optimize care for patients requiring clozapine therapy.

